# Effects of serum 25-hydroxyvitaminD level on decreased bone mineral density at femoral neck and total hip in Chinese type 2 diabetes

**DOI:** 10.1371/journal.pone.0188894

**Published:** 2017-11-30

**Authors:** Liting Guo, Zhihong Gao, Huanqi Ge

**Affiliations:** 1 Department of Endocrinology, Teda International Cardiovascular Hospital, Tianjin, China; 2 Department of Endocrinology, General Hospital of Tianjin Medical University, Tianjin, China; University of Alabama at Birmingham, UNITED STATES

## Abstract

**Objective:**

The aims of this study is to observe the levels of serum 25-hydroxyvitaminD (25OHD), parathyroid hormone and bone mineral density (BMD) in type 2 diabetes as well as to analyze the correlationship between 25OHD level and BMD.

**Methods:**

The subjects included 368 type 2 diabetic patients, ages ranged 40–79 years and 300 non-diabetic control subjects matched for age, gender and body mass index. The serum 25OHD concentration, parathyroid hormone level and BMDs value at lumbar spine (L1-L4), femoral neck, total hip and total body were measured. The BMDs (g/cm^2^) was measured by LUNAR's DEXA dual-energy X-ray absorptiometry.

**Results:**

①Compared with control subjects, the serum 25OHD level, BMDs at the femoral neck and total hip declined in type 2 diabetes[(45±17 vs. 36±12 nmol/L), (0.93±0.17 vs. 0.85±0.14 g/cm^2^), (0.93±0.14 vs. 0.87±0.15g/cm^2^) (all P<0.05)]; The parathyroid hormone level in type 2 diabetes was higher in type 2 diabetes than that in control subjects (8.5±4.2 vs. 5.6±3.9 pmol/L) (P<0.05).

②Compared with diabetes duration ≤10 years group, BMDs at the femoral neck and total hip decreased in diabetes duration >10years group [(0.88±0.11 vs. 0.81±0.15 g/cm^2^), (0.91±0.14 vs. 0.84±0.16 g/cm^2^)(All P<0.05)]; The parathyroid hormone level increased in diabetes duration >10years group than diabetes duration ≤10 years group (10.6±9.1 vs. 7.1±3.7 pmol/L) (P<0.05).

③ Compared with hemoglobin A1c (HbA1c) ≤8% group, 25OHD and BMDs at the femoral neck and total hip in HbA1c>8% group decreased [(40±15 vs. 32±13 nmol/l), (0.89±0.13 vs. 0.83±0.13 g/cm^2^), (0.95±0.13 vs. 0.83±0.16 g/cm^2^) (All P<0.05)] and the parathyroid hormone level increased (7.2±4.0 vs. 10.0±8.8 pmol/L) (P<0.05).

④The morbidity of diabetic osteoporosis and osteopenia (41.0%, 47.8%) were higher than those in control subjects (27.0%,33.3%) (*X*^*2*^ = 4.37 and 4.70, P = 0.04 and 0.03); Diabetes duration, HbA1c and parathyroid hormone levels were longer or higher in Diabetic osteoporosis group than those in normal BMD group and osteopenia group(All p<0.05).

⑤ Simple factor correlation analysis showed that the BMD at the femoral neck was negatively correlated with the age, diabetes duration, HbA1c, parathyroid hormone (rs = -0.18,-0.23,-0.18,-0.25), and positively correlated with 25OHD (rs = 0.23).

**Conclusions:**

Decreased BMDs and increased incidence of osteoporosis were observed in type 2 diabetic patients, which are closely related to the serum 25OHD level. These findings were more prominent at the femoral neck and total hip for patients with a longer diabetic history and poor glycemic control.

## Introduction

Diabetes mellitus and osteoporosis are the two most common diseases in elderly individuals [1,2]. Osteoporosis is a systemic skeletal disorder characterized by impaired bone quality and microstructural deterioration, resulting in the increased likelihood of bone fracture [3]. This is a significant social health problem for middle-age and elderly individuals. A recent study [4] reported that over 10 million adults have osteoporosis and nearly 43 million have decreased bone density. In China, the incidence of osteoporosis is approximately 20% for patients older than 40 years of age [5]. Presently, the awareness of and treatment for osteoporosis is very low, therefore research on osteoporosis is attracting more attention.

There is confirmed evidence that there is an association between diabetes and bone metabolism. The incidence of osteoporosis and fractures in diabetic (types 1 and 2) patients was higher than those in non-diabetic subjects, especially in the decreased bone mineral density (BMD) population [6–9]. Koh [10] showed that the fracture risk at the spine and hip increased 1.7–2.2-fold in type 2 diabetes compared with control subjects, and the fracture risk increased 2.5–3.4-fold when the diabetes duration was greater than 15 years.

Another risk factor for fracture may be hypovitaminosis D, as established for the general population [11–13]. Vitamin D has an important effect on bone metabolism, which is formed in the epidermis or provided by the diet. Heike [14] showed a positive association between vitamin D status and BMD at the femoral neck. Compared with subjects with vitamin D deficiency, those with vitamin D insufficient subjects had a 7.3% higher BMD and vitamin D replete subjects had a 8.5% higher BMD. In addition, vitamin D deficiency may indirectly increase risk of diabetes progression by contributing to low bone density.

Lower BMD is a primary risk factor for higher risk of fracture [14–17]. Each standard deviation of decreased BMD yields a 3-fold increase in fracture risk [18]. Previously, decreased BMD has been recognized in type 1 diabetes [19]. However, there has been controversy over its trend in type 2 diabetes. Several studies have suggested that type 2 diabetes is associated with a decreased BMD [4, 20], while other studies report a normal or increased BMD in type 2 diabetes [21–23]. This study detected the serum 25-hydroxyvitamin D concentration, parathyroid hormone level and evaluated the BMDs value at the lumbar spine (L1-L4), femoral neck, total hip and total body in all subjects to evaluate their levels in type 2 diabetes and to explore the correlativity factors of decreased BMD.

## Subjects and methods

Three hundred and sixty-eight patients with type 2 diabetes were randomly selected at the endocrinology department of General Hospital in Tianjin Medical University and Teda International Cardiovascular Hospital in Tianjin from January 2014 to June 2016. The subjects included 121 males and 247 females. The ages of subjects ranged from 40–79. At the same time, three hundred non-diabetic subjects were selected matched for age, gender, Body Mass Index (BMI) and history of smoking and alcoholism at the two hospitals mentioned above. The study protocol was approved by the Independent Ethics Committee (IEC) of Teda International Cardiovascular Hospital. All subjects gave written consent for participation. The supplementary registration institution is Chinese Clinical Trial Registry, and registration number is ChiCTR-ROC-17010468. I am sorry to say, we did not register before participant recruitment due to my work negligence.

http://dx.doi.org/10.17504/protocols.io.kjycupw[PROTOCOL DOI]; Password:15854552328

### Inclusion criteria

Type 2 diabetes mellitus conformed to the 1999 WHO Diagnostic Criteria. Non-diabetic subjects had a normal blood sugar, blood lipids and blood pressure.

### Exclusion criteria

Subjects excluded with hyperthyroidism, hyperparathyroidism and Paget’s disease. Subjects receiving corticosteroids, gonadal hormones, immunosuppressant or anticonvulsants medications were excluded from both groups. Subjects on thiazolidinediones from diabetes group were also excluded. Subjects with severe hepatopathy, nephropathy and neoplastic diseases were also excluded. Subjects did not take drugs affecting bone metabolism (such as vitamin D and its derivatives, calcium, diphosphonate) within six months. No Female subjects were pregnant or lactating.

### BMD measurements

The BMDs (g/cm^2^) at the lumbar spine (L1-L4), femoral neck, total hip, and whole body were measured by LUNAR's DEXA dual-energy X-ray absorptiometry. To eliminate technical variation, the same operator measured all subjects. The values of lumbar spine BMD were presented as the mean of the L1-L4. T scores were calculated on the basis of the normal reference values in the age and gender-matched Chinese group. Based on the WHO diagnostic criteria, patients were diagnosed with osteoporosis if their BMD was < -2.5 SD, and were diagnosed with osteopenia if their BMD was between -1.0 and -2.5 SD, and they were considered to have a normal BMD if their BMD was > - 1.0 SD.

### Grouped

Type 2 diabetic patients were divided into groups according to the diabetes duration and hemoglobin A1c (HbA1c) level: the diabetes duration ≤10 years group and the diabetes duration>10 years group; the HbA1c ≤8% group and the HbA1c >8% group.

### Biochemical measurements

All subjects were asked for details about their height and weight, and BMI was calculated. Elbow venous blood (5 ml) was collected from each subject in the morning after 8 hours of fasting. The HbA1c was measured with a TOSOH G7 analyzer, serum 25OHD was determined by enzyme immunoassay (EIA), and serum PTH was measured by a radioimmunoassay. Serum 25OHD levels less than 50 nmol/l can be defined as definitive vitamin D deficiency [24].

### Statistical analysis

Data were analyzed using SPSS 19.0 software. Quantitative data were in accordance with normal distribution. Measurement data were expressed as the ($\bar{x}\pm \text{s}$). Homogeneity of variance using ANOVA analysis. Qualitative data using the chi-square test. The BMD-related factors were analyzed by simple correlation analysis. A *P* value <0.05 was considered statistically significant.

## Results

Tables 1 and 2 **show a comparison of general data and BMDs between various groups**. There was no significant difference between diabetes and control subjects in terms of the age and BMI. The 25OHD levels and BMDs at the femoral neck and total hip were significantly lower in diabetes than those in control subjects (P<0.01 and P<0.05); however, the PTH levels increased in the diabetes compared to control subjects. Male subjects of diabetes only had a significantly decreased BMD at the femoral neck than male of control subjects, while female of diabetes had a severe decreased BMDs at the femoral neck, total hip and whole body than female of control subjects and also had a severe 25OHD deficiency (Tables 1 and 2).

**Table 1:** Comparison of general data and BMDs between various groups (${\bar{x}}^{\pm \text{s}}$).

Groups	N	Age (years)	BMI (kg/m^2^)	25OHD (nmol/L)	PTH (pmol/L)
Control subjects	300	60±12	25±5	45±17	5.6±3.9
Diabetes	368	61±8	26±5	36±12*	8.5±4.2*
Male of control subjects	83	60±11	25±5	47±16	5.2±3.9
Male of diabetes	121	60±9	27±5	36±13^#^	7.9±4.3^#^
Female of control subjects	217	58±11	24±4	44±18	5.8±3.9
Female of diabetes	247	60±8	25±5	35±12^#^	8.9±4.1^#^

BMI- Body Mass Index; 25OHD-25-Hydroxyvitamin D; PTH-Parathyroid Hormone; *P* value <0.05 is considered significant.

Control subjects vs. diabetes *P<0.01; Male of control subjects vs. male of diabetes ^#^P<0.05; Female of control subjects vs. female of diabetes ^#^P<0.05.

**Table 2:** Comparison of general data and BMDs between various groups (${\bar{x}}^{\pm \text{s}}$).

Groups	BMD (g/cm^2^)			
	lumbar spine(L1-L4)	femoral neck	total hip	whole body
Control subjects	1.10±0.21	0.93±0.17	0.93±0.14	0.93±0.14
Diabetes	1.05±0.19	0.85±0.14*	0.87±0.15^#^	0.88±0.20
Male of control subjects	1.15±0.22	1.01±0.16	0.98±0.09	0.98±0.09
Male of diabetes	1.08±0.17	0.91±0.11^#^	0.94±0.13	0.93±0.19
Female of control subjects	1.08±0.20	0.90±0.17	0.92±0.15	0.92±0.15
Female of diabetes	1.02±0.21	0.80±0.13*	0.83±0.16^#^	0.84±0.12^#^

BMD-Bone Mineral Density; *P* value <0.05 is considered significant.

Control subjects vs. diabetes *P<0.01, ^#^P<0.05; Male of control subjects vs. male of diabetes ^#^P<0.05; Female of control subjects vs. female of diabetes *P<0.01, ^#^P<0.05.

Tables 3 and 4 **show a comparison of 25OHD, PTH and BMDs among different diabetes duration groups and control group**. There was no significant difference in HbA1c level between diabetes duration ≤ 10 years group and diabetes duration > 10 years group (9.3±1.7 vs. 9.6±2.6%).

**Table 3:** Comparison of 25OHD, PTH and BMDs between different diabetes duration groups (${\bar{x}}^{\pm \text{s}}$).

Group	N	Age (years)	BMI (kg/m^2^)	25OHD (nmol/L)	PTH (pmol/L)
Control group	300	60±12	25±5	45±17	5.6±3.9
Diabetes duration≤10 years group	246	60±8	26±5	39±16	7.1±3.7
Diabetes duration>10 years group	122	62±8	25±4	34±11*	10.6±9.1*#
Value of *F*	-	4.84	1.23	5.79	10.96

BMI-Body Mass Index; 25OHD-25-Hydroxyvitamin D; PTH-Parathyroid Hormone; *P* value <0.05 is considered significant.

Compared with control group *P <0.05; compared with diabetes duration ≤10 years group ^#^P<0.05.

**Table 4:** Comparison of 25OHD, PTH and BMDs between different diabetes duration groups (${\bar{x}}^{\pm \text{s}}$).

Group	BMD (g/cm^2^)			
	lumbar spine	femoral neck	total hip	whole body
Control group	1.10±0.21	0.93±0.17	0.93±0.14	0.93±0.14
Diabetes duration≤10 years group	1.08±0.18	0.88±0.11	0.91±0.14	0.92±0.20
Diabetes duration>10 years group	1.02±0.21	0.81±0.15*#	0.84±0.16*#	0.85±0.19*
Value of *F*	2.29	9.15	6.48	3.94

BMD-Bone Mineral Density.

*P* value <0.05 is considered significant.

Compared with control subjects*P <0.05; compared with diabetes duration ≤10 years group ^#^P<0.05.

Compared with control group and diabetes duration ≤ 10 years group, 25OHD and BMDs at the femoral neck, total hip and whole body decreased in diabetes duration > 10 years group (all *P*< 0.05), especially BMDs at the femoral neck and total hip decreased more significantly. On the contrary, PTH level increased significantly in diabetes duration >10 years group than that in control group and diabetes duration ≤10 years group(Tables 3 and 4).

Tables 5 and 6 **show a comparison of 25OHD, PTH and BMDs among different HbA1c level groups and control group**. No significant difference was found in diabetes duration between HbA1c ≤ 8% and HbA1c > 8% groups (10.0±5.2 vs. 10.5±6.0).

**Table 5:** Comparison of 25OHD, PTH and BMDs between different HbA1c groups (${\bar{x}}^{\pm \text{s}}$).

Group	N	Age (years)	BMI (kg/m^2^)	25OHD nmol/L	PTH pmol/L
Control group	300	60±12	25±5	45±17	5.6±3.9
HbA1c≤8% group	184	60±8	26±5	40±15	7.2±4.0
HbA1c>8% group	184	62±9	25±4	32±13*^#^	10.0±8.8*^#^
Value of *F*		4.12	1.24	8.26	9.18

BMI- Body Mass Index; 25OHD-25-Hydroxyvitamin D; PTH-Parathyroid Hormone; *P* value <0.05 is considered significant.

Compared with the control group *P<0.05; compared with the HbA1c ≤8% group ^#^P<0.05.

**Table 6:** Comparison of 25OHD, PTH and BMDs between different HbA1c groups (${\bar{x}}^{\pm \text{s}}$).

Group	BMD (g/cm^2^)			
	lumbar spine(L1-L4)	femoral neck	total hip	whole body
Control group	1.10±0.21	0.93±0.17	0.93±0.14	0.93±0.14
HbA1c≤8% group	1.04±0.19	0.89±0.13	0.95±0.13	0.89±0.15
HbA1c>8% group	1.05±0.20	0.83±0.13*^#^	0.83±0.16*^#^	0.86±0.21*
Value of *F*	1.15	5.38	3.04	1.64

BMD-Bone Mineral Density; *P* value <0.05 is considered significant.

Compared with the control group *P<0.05; compared with the HbA1c ≤8% group ^#^P<0.05.

Compared with control group and HbA1c ≤ 8% group, 25OHD and BMDs at the femoral neck, total hip and whole body were significantly lower in HbA1c > 8% group (all *P*< 0.05). But PTH increased significantly in HbA1c >8% group(Tables 5 and 6).

Table 7 **shows a comparison of the proportions of osteoporosis, osteopenia and normal BMD between diabetes group and control group**. The proportions of osteoporosis and osteopenia were 41.0% and 47.8% in diabetes group, which were significantly higher than 27.0% and 33.3% in control group (P<0.05). The proportion of normal BMD was only 11.1% in the diabetes group, while it was 39.7% in the control group (Table 7).

**Table 7:** Comparison of the proportion of osteoporosis, osteopenia and normal BMD between diabetes group and control group.

Group	Osteoporosis	Osteopenia	Normal BMD	
Control group	81(27.0%)	100(33.3%)	119(39.7%)	*P* = 0.00
Diabetes group	151(41.0%)	176(47.8%)	41(11.2%)	*X*^*2*^ = 22.15
Value of *X*^*2*^	4.37	4.70	38.93	
Value of *P*	0.04	0.03	0.00	

P value <0.05 is considered significant

Table 8 **shows a comparison of all indexes at different BMD subgroups in diabetic patients**. The levels of PTH, HbA1c and diabetes duration were all significantly higher in osteoporosis group than osteopenia group and normal BMD group (P<0.05), whereas 25OHD also presented a downward trend in osteopenia group and osteoporosis group (Table 8).

**Table 8:** Comparison of all indexes at different BMD subgroups in diabetic patients.

Group	N	25OHD (nmol/L)	PTH (pmol/L)	HbA1c (%)	Diabetes duration (years)
Normal BMD group	41	41±18	7.2±3.7	8.3±2.2	9.2±6.7
Osteopenia group	176	34±13	7.5±2.7	8.7±2.0	11.0±6.5
Osteoporosis group	151	35±13	10.8±3.2*^#^	10.0±2.8*^#^	14.9±7.3*^#^

HbA1c-Hemoglobin A1c; PTH-Parathyroid Hormone; *P* value <0.05 is considered significant.

Compared with the normal BMD group *P <0.05; compared with the osteopenia group ^#^P <0.05

Table 9 **shows a simple correlation analysis of the femoral neck BMD**. The above results showed that the femoral neck BMD decreased the most sensitively; therefore, a simple correlation analysis was performed with the femoral neck BMD as the dependent variable, and diabetes duration, HbA1c, PTH and 25OHD levels as the independent variables. The results revealed that BMD at the femoral neck was negatively correlated with diabetes duration, HbA1c and PTH, whereas it was positively correlated with 25OHD (Table 9).

**Table 9:** A single factor correlation analysis of the femoral neck BMD.

correlation factors	femoral neck	
	rs	P
Age(years)	-0.18	0.04
Diabetes duration(years)	-0.23	0.008
HbA1c(%)	-0.18	0.042
PTH(pmol/l)	-0.25	0.006
25OHD (nmol/l)	0.23	0.008

rs-correlation coefficient; HbA1c-Hemoglobin A1c; 25OHD-25-Hydroxyvitamin D; PTH-Parathyroid Hormone; P value <0.05 is considered significant

## Discussion

Recently, Diabetes and osteoporosis are the two most common diseases in elderly individuals [4.10.20]. A lower BMD is a risk factor for osteoporosis and bone fractures [14]. The BMD level, however, is not fully understood, and there remains controversy over its value in diabetes. One study [22] reported that the BMD of the hip is lower in diabetes than in age and BMI-matched non-diabetes men. Another study [19] demonstrated that the BMD was lower in diabetic group than healthy control group, and a negative correlation was observed between the BMDs and both the serum glucose level and HbA1c. Our study found that the BMDs at the femoral neck and total hip were significantly lower in diabetic patients than those in control subjects, especially among women. However, male diabetic patients only presented with a significantly decreased BMD at the femoral neck. The proportions of osteoporosis and osteopenia were significantly higher in diabetes group than control subjects. Nearly half of diabetic patients had osteoporosis, whereas the proportions of osteoporosis plus osteopenia were as high as 88.8%. Our study showed that, there were a higher incidence of osteoporosis and a lower BMD value in Chinese type 2 diabetes, which should be aroused our attention. Similarly, an 11-year observational study [24] of 32,089 postmenopausal diabetic women demonstrated significantly decreased BMDs at the femoral neck and hip as well as significantly increased incidence of hip fracture in diabetic patients, which was 1.7 times that of the non-diabetes group. In another cross-sectional study of American men [25], comparison of the BMD between 3,458 non-diabetic controls and 735 type 2 diabetic patients also showed a significantly lower hip BMD and increased osteoporosis incidence in type 2 diabetes group than non-diabetes group. According to a 5-year follow-up longitudinal study of diabetic women without a history of osteoporosis by Hamilton et al. [26], showed that the BMD at all parts was significantly reduced compared with the BMD five years prior, except for the lumbar spine BMD. The above three studies [24–26] showed an unchanged or increased lumbar spine BMD. The lumbar spine BMD was mostly unchanged or increased, which may be due to the lumbar vertebra propensity to hyperostosis, especially the vertebral edge hyperplasia and even bone bridge formation in the elderly which led to the measurement of false normal or lumbar spine BMD increased.

The influence of diabetes duration and HbA1c on the BMD were also observed in this study. Diabetes duration > 10 years group and HbA1c > 8% group had a significantly lower BMDs at the femoral neck and total hip than diabetes duration ≤ 10 years group and HbA1c ≤ 8% group, suggesting that there was a substantial influence of diabetes duration and control level of blood glucose on the BMD. Consistent with the foreign studies, to assess the BMD levels of type 2 diabetes, Majima [27] compared the BMDs of the cortical bone (distal radius) and cancellous bone (lumbar spine and femoral neck) and Z scores between 145 type 2 diabetes and 95 controls, which showed that the average HbA1c level was negatively correlated with the male and female proximal radius and the female femoral neck BMD. However, Zhang et al.'s report [28] found no correlation between the BMD and HbA1c in male diabetic patients. For each additional 15 years of diabetes duration, the BMD will be reduced by 10% according to Kown et al [29]. Janghorbani et al [30] reported that the longer the duration of diabetes, the higher the risk of hip fracture; in addition, for type 2 diabetes duration ≥ 10 years, the risk of hip fracture was 2.7 times that of the non-diabetes group. We also compared the diabetes duration and HbA1c level at different bone mass distributions, which revealed that the average diabetes duration and HbA1c reached 14.9 years and 10.0%, respectively, in diabetic osteoporosis group, which were significantly higher than osteopenia group and normal BMD group. Additionally, we performed a single factor correlation analysis of the femoral neck BMD and found that the diabetes duration and HbA1c were both negatively correlated with the femoral neck BMD.

It is well known that the important factors influencing BMD level are 25OHD, PTH hormone level. In this study, a significantly lower 25OHD level was observed in diabetics group than those in control group. As the researches abroad [31] the 25OHD level was significantly lower in diabetes duration > 10 years group and HbA1c > 8% group than that in control group. Correlation analysis showed a positive correlation between the 25OHD level and BMD, consistent with the foreign research [14, 32]. Bischoffferrari, et al[14] also observed a significant positive association between serum 25OHD and BMD in individuals with primary knee OA. Akhter et al [33] analyzed 112 African American men for the 25OHD level and BMD and found that those with serum 25OHD levels lower than 10 ng/ml and 30 ng/ml accounted for 24% and 89%, respectively; in addition, when 25OHD_3_ was lower than 15 ng/ml, the 25OHD level was positively correlated with the BMD. Given the high prevalence of low 25OHD status in diabetes, and the positive association between 25OHD and BMD, vitamin D supplementation may enhance BMD in individuals with diabetes. One classical pathway of CYP11A1 to metabolize D3 was: D3→25OHD→1,25(OH)_2_D3. Recent study showed that skin cells can metabolize D3 through the novel CYP11A1 initiated pathway, as an additional and alternative pathway to the classical one, where showed a slightly higher production of 1,25(OH)2D3 to improve Vitamin D level[34,35]. The novel pathways of D3 metabolism initiated by CYP11A1 and modified by CYP27B1 activity, these findings define the pathway intermediates (include 20(OH)D3, 22(OH)D3, 20,23(OH)(2)D3, 1,20(OH)(2)D3 etc.) as natural products/endogenous bioregulators and break the current dogma that vitamin D is solely activated through the sequence D3 → 25(OH)D3 → 1,25(OH)(2)D3[36,37].

Research on the action of vitamin D (VitD) on bone mechanisms has been increasing; in addition to having classic effects on bone, recent studies have shown that VitD also has a role in bone formation as well as a facilitating role in collagen generation and osteoblasts. Bone formation decreased under the diabetes state, while bone resorption increased compared to bone formation; furthermore, in the diabetes population, vitamin D receptor (VDR) gene polymorphism was also closely associated with BMD [38]. Vitamin D insufficiency is associated with increased PTH levels, accelerated cortical bone loss and an increased risk of fractures, and concluded the relationship between PTH and 25OHD decreases smoothly [39]. In our study, PTH was significantly higher in diabetes group than control group, and it was significantly higher in diabetic osteoporosis group than osteopenia group and normal BMD group. Correlation analysis demonstrated that PTH level was negatively correlation with the femoral neck BMD. Among 368 type 2 diabetes, 70 (59.8%) had an elevated PTH level. The study by Nagasaka et al [40] showed that long-term hyperglycemia in type 2 diabetes leads to hypocalcemia, and stimulating PTH secretion, which may be the cause of osteopenia among diabetic patients. The improvement of glucose metabolism may be somewhat helpful in reducing the PTH level. Chen et al [41] found that the rate of PTH level increased was over 30% among elderly fracture patients, but they did not find any correlation between PTH level and BMD. PTH secretion can be directly regulated by the VitD level and blood calcium. In the earlier period, PTH elevation may be stimulated by a lower VitD level and lower blood calcium and it would reduce bone formation. In later period, increased PTH further enhances osteoclast activity, leading to increased bone resorption and further reduction of the BMD.

In summary, the BMD is influenced by many factors, such as genetic factors and non-genetic factors, especially geographic and environmental factors; changes in diet and exercise; nutritional, obesity and metabolic changes; medication, etc. The timely detection of serum 25OHD level and BMD should be taken seriously. Patients with a diabetes duration > 10 years and consistent HbA1c > 8% and who are over 50 years of age should be evaluated for their BMD and 25OHD level as early as possible. Numerous studies have demonstrated the non-negligible influence of diabetes mellitus on BMD, including diabetes duration and glycemic control level. Strict glycemic control is also very necessary for preventing osteoporosis and fractures. Vitamin D supplementation may enhance BMD in individuals with diabetes. Some study suggested that a high dose daily intake of 4000IU vitamin D to maintain 25OHD ranged from 100–150 nmol/l. This study was limited by the smaller sample size; we would increase the sample to have a vitamin D supplementation dose in the next study.

## Supporting information

S1 Appendix Basic information of all subjects. (XLS)

S2 Appendix CONSORT checklist. (DOC)

S3 Appendix Trial protocol. (DOC)

S4 Appendix Consent form. (DOC)

S5 Appendix Case report form. (DOC)

S1 Fig Flowchart of participates’ progress through the phases of the trial. (DOCX)
