# Supplementary material for: Effects of serum 25-hydroxyvitaminD level on decreased bone mineral density at femoral neck and total hip in Chinese type 2 diabetes
Source: PLoS One. 2017 Nov 30;12(11):e0188894. doi: 10.1371/journal.pone.0188894 (PMC5708672; doi:10.1371/journal.pone.0188894)
Supplement: S3 Appendix — (DOC) [file pone.0188894.s003.doc]

***Effects of serum 25-HydroxyvitaminD level on decreased bone mineral density at femoral neck and total hip in Chinese type 2 diabetes***

**Detailed study protocol**

**13 January 2014**

**ASSET Research Team**

Research leader, Study Design and statistical analysis: Teda International Cardiovascular Hospital

Project contact: Liting Guo Tel:13820797731

Data collection and technology implementation: Teda International Cardiovascular Hospital

Project contact: Huanqi Ge Tel: 022-65208072

Data collection and Research manager: General Hospital of Tianjin Medical University

Project contact: Zhihong Gao Tel:13662041912

Contents

[1. Background 2](#__RefHeading___Toc250301980)

[2. *St*udy Design 3](#__RefHeading___Toc250301981)

[3. Ethics 4](#__RefHeading___Toc250301984)

[4. Eligibility 4](#__RefHeading___Toc250301985)

[5. Recruitment 5](#__RefHeading___Toc250301986)

[6. Baseline and repeated assessments 5](#__RefHeading___Toc250301987)

[7. Diagnosed and study group assignment 5](#__RefHeading___Toc250301988)

[8. Data measurment 6](#__RefHeading___Toc250301986)

[9. Data Handling and Record keeping 7](#__RefHeading___Toc250301987)

[10. Statistical Methods 8](#__RefHeading___Toc250301988)

[11. Timeline 9](#__RefHeading___Toc250301998)

[Appendix 1. Baseline data collection](#__RefHeading___Toc250302000) [1](#__RefHeading___Toc250302003)0

[Appendix 2. Study flow diagrams 11](#__RefHeading___Toc250302004)

[Appendix 3.Case report form](#__RefHeading___Toc250302005) [1](#__RefHeading___Toc250302006)2

List of acronyms and abbreviations

HbA1c Glycosylated hemoglobin A1c

BMD Bone mineral density

PTH Parathyroid hormone

T2DM Type 2 Diabetes

25OHD 25-hydroxyvitamin D

OP Osteoporosis

BMI Body mass index

***Research Protocol of* *Effects of serum 25-HydroxyvitaminD level on decreased bone mineral density at femoral neck and total hip in Chinese type 2 diabetes***

**1. Background**

Diabetes and osteoporosis are the two most common diseases in elderly individuals . Osteoporosis is a systemic skeletal disorder characterized by impaired bone quality and microstructural deterioration, resulting in the increased likelihood of bone fracture . This is a significant social health problem for middle-age and elderly individuals. A recent study reported that over 10 million adults have osteoporosis and nearly 43 million have decreased bone density. In China, the incidence of osteoporosis is approximately 20% for patients older than 40 years of age, and it is approximately 20.7% at the vertebral body and femoral neck for female patients older than 50 years of age. Presently, the awareness of and treatment for osteoporosis is very low; therefore, research on osteoporosis is attracting more attention.

There is increasing evidence confirming that there is an association between diabetes and bone metabolism. The latest research showed that the prevalence of diabetes is 11.6% in Chinese adults, accounting for approximately 110 million people. The incidence of osteoporosis and fractures in diabetes (types 1 and 2) patients was higher than in non-diabetes patients, especially in the decreased bone mineral density (BMD) population. Koh showed that the fracture risk at the spine and hip increased 1.7-2.2-fold in T2DM patients compared with control subjects, and the fracture risk increased 2.5-3.4-fold when the disease duration was greater than 15 years.

The BMD has been identified as a crucial deciding predictor of osteoporosis and osteoporotic fractures, and it is more serious in diabetic conditions. Previously, decreased BMD has been recognized in type 1 diabetes. However, there has been controversy over its trend in type 2 diabetes. Several studies have suggested that type 2 diabetes is associated with a decreased BMD, while other studies report a normal or increased BMD in type 2 diabetes. This study detected the serum concentration of 25-hydroxyvitamin D and Parathyroid hormone and evaluated the BMD at the lumbar spine (L1-L4), femoral neck, total hip and total body in all subjects. This study also explored their variation tendency and analyzed BMD-related factors.

# 2. Study Design

It will be conducted in two study sites (General Hospital of Tianjin Medical University and Teda International Cardiovascular Hospital). Each study site involves a distinct population group and service model though share some outcomes . The baseline information (Appendix1) were filled by diabetic patients who participated in weekly continuing Outpatient follow-up between 13^th^January 2014 and 1^th^June 2016 , in Tianjin China. The patients were randomly selected from outpatient that provide Comprehensive information according to Appendix1.

The BMD equipment will be available to all services for two and a half years, To eliminate technical variation, the same operator measured all subjects.

Subjects were assessed for eligibility from January 13th, 2014 to June1th, 2016.

Standard criteria and measures will apply across all trials.

# 3. Ethics

The Independent Ethics Committee (IEC) of Teda International Cardiovascular Hospital.

No patients will be enrolled before ethics approval is obtained.

All subjects gave informed consent for participation.

A researcher will provide an approved informed consent for participation to all people who are potentially eligible to participate. Subjects who provide written consent will have baseline data collected. From those who do not consent to participate we will seek to obtain basic demographic information.

# 4.Eligibility

**Inclusion criteria(Appendix3):**

a. age 40-79 years old

b. living permanently at home

c. able to communicate in Chinese (or someone in household able to translate)

d. able to accept the determined of BMD equipment

e. conformed to diagnostic criteria for Diabetes Mellitus

f. gave informed consent for participation

**Exclusion criteria:**

a. Severe liver, kidney and neoplastic diseases

b.Patients who participated in other clinical trials in the past 30 days d. accept glucocorticoid therapy

c. patients suffered from Diabetic ketoacidosis

d. Pregnant women

e. patients suffered from thyroid disease

f. patients suffered from parathyroid disease

g. patients suffered from adrenal disease

h. patients suffered from gonadal disease

I. patients suffered from pituitary diseases

j. Patients who taking drugs affecting bone metabolism (such as steroids, VitD and its derivatives, calcium, diphosphonate and thiazolidinedione antidiabetics)

k. patients who cannot sign informed consent

# 5. Recruitment

**Numbers potentially available**

Randomly selected 200 known patients with Diabetes from General Hospitan of Tianjin Medical University - About 2000 diabetes are admitted to hospital each year

Randomly selected 200 known patients with Diabetes Teda International Cardiovascular Hospital - about 1500 patients are admitted to hospital each year;

At same time selected 312 normal controls randomly (Appendix2).

**Recruitment strategies:**

Identified by WHO1999S on admission with Diabetes,screened for eligibility and consent to pass name to researcher, who will conduct formal consent process.

# 6.Baseline and repeated assessments

All study participants, in diabetes and control groups, will have the same data collected relating to quality of life and resource use at baseline

Those in the diabetes group will be asked additional questions relating to the fasting glucose, HbA1c, diabetes duration.

For baseline data collection see **Appendix 1**.

# 7. Diagnosed and study group assignment

Based on the WHO diagnostic criteria, patients were diagnosed with osteoporosis if their BMD was < -2.5 SD, diagnosed with osteopenia if their BMD was between -1.0 and -2.5 SD, and they were considered to have a normal BMD if their BMD was > - 1.0 SD.

a. WHO (1999) diagnostic criteria for diabetes

1. Diabetes symptoms + plasma glucose level at any time ≥11.1 mmol/l (200 mg/dl) or

2. Fasting plasma glucose (FPG) level ≥ 7.0 mmol/l (126 mg/dl)

3. OGTT test showing 2-hour PG level ≥ 11.1mmol/l (200 mg/dl)

Immediately after baseline data collection, participants will be grouped to different sub-groups or control group.

Participants with T2DM were divided into groups according to the diabetes duration and HbA1c level: diabetes duration≤10years, diabetes duration>10years; HbA1c≤8%, and HbA1c>8% group.

Eligible 🡪 consent 🡪 baseline data collection 🡪 individual randomly assigned to sub-diabete groups or control

**8.Data measurement**

1. Basic information collected(Appendix1)

2. BMD measured by LUNAR's DEXA dual-energy X-ray absorptiometry(table1)

3. Laboratory examination (table1)

Table1 Each subject need to be determined Laboratory projects and BMDs at different sites

**Laboratory examination Date**

Project Measured value

HbA1c

25-hydroxyvitamin D

Parathyroid hormone

Bone mineral density (kg/m2)

lumbar spine（L1-L4）

Femoral neck

Total hip

Total body

*Primary quantitative health outcomes and resource use*

The serum 25-hydroxyvitaminD level was significantly decreased and the parathyroid hormone level was significantly increased in type 2 diabetes patients than those in control subjects, and the BMD at the femoral neck and total hip in T2DM patients was much lower than that in control subjects. In parallel with the diabetes duration, BMDs at the femoral neck and total hip decreased, and parathyroid hormone increased. Moreover, the BMDs at the femoral neck, total hip and total body decreased in patients with poor glycemic control. In addition, the proportions of patients with osteoporosis and osteopenia were significantly higher in T2DM patients than in control subjects. There may be a negative correlation between the BMD and diabetes duration as well as the HbA1c and parathyroid hormone, and there may be a positive correlation between the BMD and 25-hydroxyvitaminD.

# 9.Data Handling and Record Keeping

**Confidentiality**

Information about study participants will be kept confidential and managed according to the requirements of The Chinese Clinical Trial, and the Ethics approval specific to this study.

**Source Documents**

Source data is all information, original records of clinical findings, observations, or other activities in a clinical trial necessary for the reconstruction and evaluation of the trial. Source data are contained in source documents. Examples of these original documents and data records include: hospital records, clinical and office charts, completed interview schedules, and subject files involved in the clinical trial.

**Case Report Forms**

The study case report form (CRF) is the primary data collection instrument for the study. All data requested on the CRF must be recorded. All missing data must be explained. If a space on the CRF is left blank because the procedure was not done or the question was not asked, write “N/D”. If the item is not applicable to the individual case, write “N/A”. All entries should be printed legibly in black ink. If any entry error has been made, to correct such an error, draw a single straight line through the incorrect entry and enter the correct data above it. All such changes must be initialled and dated. **Do not erase or white-out errors.** For clarification of illegible or uncertain entries, print the clarification above the item, then initial and date it.

**Records Retention**

It is the local researcher’s responsibility to retain study essential documents and deliver these to the Study Manager. The Study Manager will ensure these records are retained securely for a period of 2.5 years from collection, or otherwise as indicated in the Ethnics approval specific to this study.

**Data and document handling**

The following table outlines the flow of information from recruitment to assessment.

Table 2: summary of data and document handling

| **Record** | **Data handling procedure** |  |
| --- | --- | --- |
| Eligible clients list | Stored electronically by researcher leader |  |
| Ineligible clients list | Stored electronically by researcher leader |  |
| Contact list | Stored electronically by Contact list |  |
| Declined clients list | Stored electronically by Declined clients list |  |
| Randomisation list | Patients randomization selected, records on Care Report Form, updates local list of Intervention and Control patients. |  |
| Enrolled participant list | Researcher maintains up-to-date list |  |
| Participant contact card | Researcher maintains up-to-date list |  |
| Withdrawn participant list | Researcher maintains up-to-date list |  |

# 10.Statistical Issues

Quantitative data were described by means and standard deviations, and A comparison of the means between groups was performed using a *t* test; a multi-group comparison of means was performed using a one-way ANOVA; the osteoporosis positive rates were compared with the *X^2^* test; and BMD-related factors were analyzed by simple correlation analysis . A *P* value <0.05 was considered statistically significant.

**Sample size**

Each region will be a stand-alone and discrete trial though with some shared outcomes. Standard criteria and measures will apply across all trials. A sample size of 200 within General Hospital of Tianjin Medical Univercity, Similarly, 200 diabetes patients within Teda International Cardiovascular Hospital to have 90% power (alpha 0.05) to detect a mean change of 2 (the minimum important difference) on the Questionnaire. Detecting some other outcomes will require more patients.

# 11.Timeline

| ID | Name | Start | Finish |
| --- | --- | --- | --- |
| 1 | Planning and ethics |  |  |
| 2 | Detailed protocol | Jan 2014 |  |
| 3 | Development of interviews and questionnaires | Jan 2014 |  |
| 4 | Ethics application | Jan 2014 |  |
| 5 | Recruitment of staff | Jan 2014-Jun 2016 |  |
| 6 | Written informed consent |  |  |
| 7 | Filled in base information |  |  |
| 8 | Data collection |  |  |
| 9 | First rollout |  |  |
| 10 | Analysis and reports |  |  |
| 11 | Data analysis |  |  |
| 12 | Draft report |  |  |
| 13 | Final report |  |  |

**Appendix1. Baseline data collection**

**Patients and family**

Date of birth, age, Gender

Height, weight, calculated body mass index

Fasting glucose, HbA1c, diabetes duration

Blood pressure

Address, Contact details

Other morbidities

Medication situation

Random assignment group

**Appendix 2. Study flow diagrams**

**Appendix3. Case Report Form**

***Effects of serum 25-HydroxyvitaminD level on decreased bone mineral density at femoral neck and total hip in Chinese type 2 diabetes***

**Case Report Form， CRF**

Trial version：1.0

Subject Name (initials)：□□□□

Number of subjects：□□□

Study leader：Liting Guo

Date：2014.1.3

Applicant's institution：Teda International Cardiovascular Hospital

**Item**

Sex Male □ Female□

Age（40-79years old） Yes □ No□

Height (cm)

Weight (kg)

BMI (kg/m^2)

Diabetes duration (years)

History of diabetes treatment

Co-morbidity

**Inclusion Criteria**  Yes No

Age:40-79 years old □ □

Conformed to diagnostic criteria for Diabetes Mellitus

□ □

Gave informed consent for participation □ □

When any of the above questions are answered "no", the case cannot be included in the study

**Exclusion criteria**

Severe liver, kidney and neoplastic diseases □ □

Patients who participated in other clinical trials in the past 30 days □ □

Glucocorticoid therapy □ □

Diabetic ketoacidosis □ □

Pregnant women □ □

Patients with thyroid disease □ □

Patients with parathyroid disease □ □

Patients with adrenal disease □ □

Patients with gonadal disease □ □

Patients with pituitary diseases □ □

Patients who taking drugs affecting bone metabolism (such as steroids, VitD and its derivatives, calcium, diphosphonate and thiazolidinedione antidiabetics)

□ □

Patients who cannot sign informed consent □ □

When any of the above questions are answered "yes", the case cannot be included in the study

**Laboratory examination Date**

Project Measured value

HbA1c

25-hydroxyvitaminD (nmol/l)

Parathyroid hormone (pmol/l)

Bone mineral density (kg/m^2)

lumbar spine（L1-L4）

Femoral neck

Total hip

Total body

The results of the above examination are required to be measured
