# Supplementary material for: Effects of serum 25-hydroxyvitaminD level on decreased bone mineral density at femoral neck and total hip in Chinese type 2 diabetes
Source: PLoS One. 2017 Nov 30;12(11):e0188894. doi: 10.1371/journal.pone.0188894 (PMC5708672; doi:10.1371/journal.pone.0188894)
Supplement: S4 Appendix — (DOC) [file pone.0188894.s004.doc]

**知情同意书**

**课题名称：**2型糖尿病患者骨密度的变化及相关影响因素

**方案版本号：**版本号：1.0，版本日期：2014年1月3日

**知情同意书版本号：**版本号：1.0，版本日期：2014年1月3日

**课题负责人：**郭丽婷

您将被邀请参加一项临床试验。本知情同意书提供给您一些信息以帮助您决定是否参加此项临床试验。请您仔细阅读，如有任何疑问请向负责该项试验的研究者提出。

您参加本项试验是自愿的。本次试验已通过本研究机构伦理审查委员会审查。

试验背景：

随着全球老龄化社会到来，骨质疏松的发病率有迅速增加的趋势，这将严重影响着全球数千万患者的身体健康及生活质量，已成为全球热点问题。众多研究表明,糖尿病(DM)患者存在骨代谢紊乱，OP和骨折的发生率增加，导致残疾率和死亡率的增高。但至今T2DM与骨密度(BMD)关系的研究仍较匮乏，本研究观察T2DM患者BMD变化，探讨T2DM病程和HbA1c对BMD的影响，分析BMD的相关因素。

试验目的：

观察T2DM患者钙调激素和骨密度(BMD)的变化,分析其相关因素。

试验简介：

随机选取T2DM患者368例,男52例,女65例,另选年龄、性别相匹配的对照(NC)组300例。测定所有受试者血清25（OH）D3、甲状旁腺激素，及腰椎（L1-L4）股骨颈、全髋、全身部位骨密度。分析不同病程组即≤10年组和>10年组，不同糖化血红蛋白(HbA1c)组即≤8%组和>8%组的血清25羟维生素D3、甲状旁腺激素水平和腰椎(L1-L4)、股骨颈、全髋及全身BMD值。

试验过程：（包括主要试验内容、试验过程与期限、随访的次数、需何种检查操作及次数、告知受试者可能被分配到试验的不同组别、用药方法等――语言要求通俗易懂）。

随机选自2014年1月至2016年6月天津医科大学总医院内分泌科和泰达国际心血管病医院门诊及住院T2DM患者368例为研究对象，男165例，女203例，年龄40-79岁，平均年龄 60.5±7.8岁，均

符合1999WHO糖尿病诊断标准。

排除标准：患有甲状腺、甲状旁腺、肾上腺、性腺及垂体疾病者，严重肝、肾疾病，肿瘤疾病者，服用影响骨代谢药物者（如类固醇激素、VitD及其衍生物、钙、双磷酸盐、噻唑烷二酮类降糖药物等）。另选取年龄和体重指数相匹配的健康对照组63例，平均年龄58.9±12.0岁，血糖、血脂、血压均正常。

分组：骨质疏松根据WHO诊断标准：骨密度低于-2.5SD为骨质疏松,(-1.0～-2.5)SD为骨量减少,>-1.0SD为骨密度正常。分析不同病程组即≤10年组和>10年组，不同糖化血红蛋白(HbA1c)组即≤8%组和>8%组的血清25羟维生素D3、甲状旁腺激素水平和腰椎(L1-L4)、股骨颈、全髋及全身骨密度值。

详细询问所有研究对象身高、体重，计算体重指数。所有受试者均于晨起空腹8小时后，取肘静脉血2ml，HbA1c由TOSOH公司G7型仪器测定，血清25(OH)D3用酶免疫分析法(EIA)测定，血清PTH用免疫放射法测定。骨密度(g/cm2)采用LUNAR公司的DEXA双能X线骨密度仪测定。

风险与不适：本次课题对研究对象无任何风险及不适症状。

潜在受益：本试验通过观察2型糖尿病患者骨密度下降程度，及与骨密度下降密切相关因素，可能会治愈糖尿病骨质疏松或阻止 / 减缓疾病的发展。尽管参加本次试验可能不会给您带来直接的益处，但您的参与可能会给未来遭受同样痛苦的患者带来益处。

费用：试验过程中的检查费自理。

报酬：参与本次试验，您不会得到报酬

赔偿：依照中国的法律

本次试验之外的备选疗法：

如果您因参与这项试验而受到伤害：如发生与临床试验相关的损害时，您可以获得免费治疗和／或相应的补偿。

作为研究受试者，您有以下职责：提供有关自身病史和当前身体状况的真实情况；不得服用受限制的药物、食物等；告诉研究医生自己在最近是否曾参与其他研究，或目前正参与其他研究。

隐私问题：如果您决定参加本项试验，您参加试验及在试验中的个人资料均属保密。负责研究医师及其他研究人员将使用您的医疗信息进行研究。这些信息可能包括您的姓名、地址、电话号码、病史及在您研究来访时得到的信息。您的档案将保存在有锁的档案柜中，仅供研究人员查阅。研究中会用编号来标识您的研究信息和实验室检查标本。只有研究者和研究小组成员可查询编号。为确保研究按照规定进行，必要时，研究者，伦理审查委员会的成员按规定可以在研究单位查阅您的个人资料。这项研究结果发表时，将不会披露您个人的任何资料。

您可以选择不参加本项试验，或者在任何时候通知研究者后退出而不会遭到歧视或报复，您的任何医疗待遇与权益不会因此而受到影响。

如果您需要其它治疗，或者您没有遵守研究计划，或者发生了与试验相关的损伤或者有任何其它原因，研究医师可以终止您继续参与本项研究。

您可随时了解与本研究有关的信息资料和研究进展，如果您有与本试验有关的问题，或您在研究过程中发生了任何不适与损伤，或有关于本项研究参加者权益方面的问题您可以通过 13820797731 （电话号码）与 郭丽婷 （研究者姓名）联系。

同意参加本试验签字： 联系电话：
