# Supplementary material for: Effects of serum 25-hydroxyvitaminD level on decreased bone mineral density at femoral neck and total hip in Chinese type 2 diabetes
Source: PLoS One. 2017 Nov 30;12(11):e0188894. doi: 10.1371/journal.pone.0188894 (PMC5708672; doi:10.1371/journal.pone.0188894)
Supplement: S5 Appendix — (DOC) [file pone.0188894.s005.doc]

**Decreased bone mineral density at the femoral neck and total hip positively correlate with 25-hydroxyvitamin D_3_ in Chinese patients with type 2 diabetes**

**Case Report Form， CRF**

Trial version：1.0

Subject Name (initials)：□□□□

Number of subjects：□□□

Study leader：Liting Guo

Date：2014.1.3

Applicant's institution：Teda International Cardiovascular Hospital

**Item**

Sex Male □ Female□

Age（40-79years） Yes □ No□

Height (cm)

Weight (kg)

BMI (kg/m^2)

Diabetic duration (years)

History of diabetes treatment (years)

Co-morbidity

**Inclusion Criteria**  Yes No

Age:40-79 years old □ □

Conformed to diagnostic criteria for Diabetes Mellitus

□ □

Gave informed consent for participation □ □

When any of the above questions are answered "no", the case cannot be included in the study

**Exclusion criteria**

Severe liver, kidney and neoplastic diseases □ □

Patients who participated in other clinical trials in the past 30 days □ □

Glucocorticoid therapy □ □

Diabetic ketoacidosis □ □

Pregnant women □ □

Patients with thyroid disease □ □

Patients with parathyroid disease □ □

Patients with adrenal disease □ □

Patients with gonadal disease □ □

Patients with pituitary diseases □ □

Patients who taking drugs affecting bone metabolism (such as steroids, VitD and its derivatives, calcium, diphosphonate and thiazolidinedione antidiabetics)

□ □

Patients who cannot sign informed consent □ □

When any of the above questions are answered "yes", the case cannot be included in the study

**Laboratory examination Date**

Project Measured value

HbA1c (%)

25OHD (nmol/l)

Parathyroid hormone (pmol/l)

Bone mineral density (kg/m2)

lumbar spine（L1-L4）

Femoral neck

Total hip

Total body

The results of the above examination are required to be measured
