# Supplementary material for: Effects of serum 25-hydroxyvitaminD level on decreased bone mineral density at femoral neck and total hip in Chinese type 2 diabetes
Source: PLoS One. 2017 Nov 30;12(11):e0188894. doi: 10.1371/journal.pone.0188894 (PMC5708672; doi:10.1371/journal.pone.0188894)

Figure1: Flowchart of participates′ progress through the phases of the trial

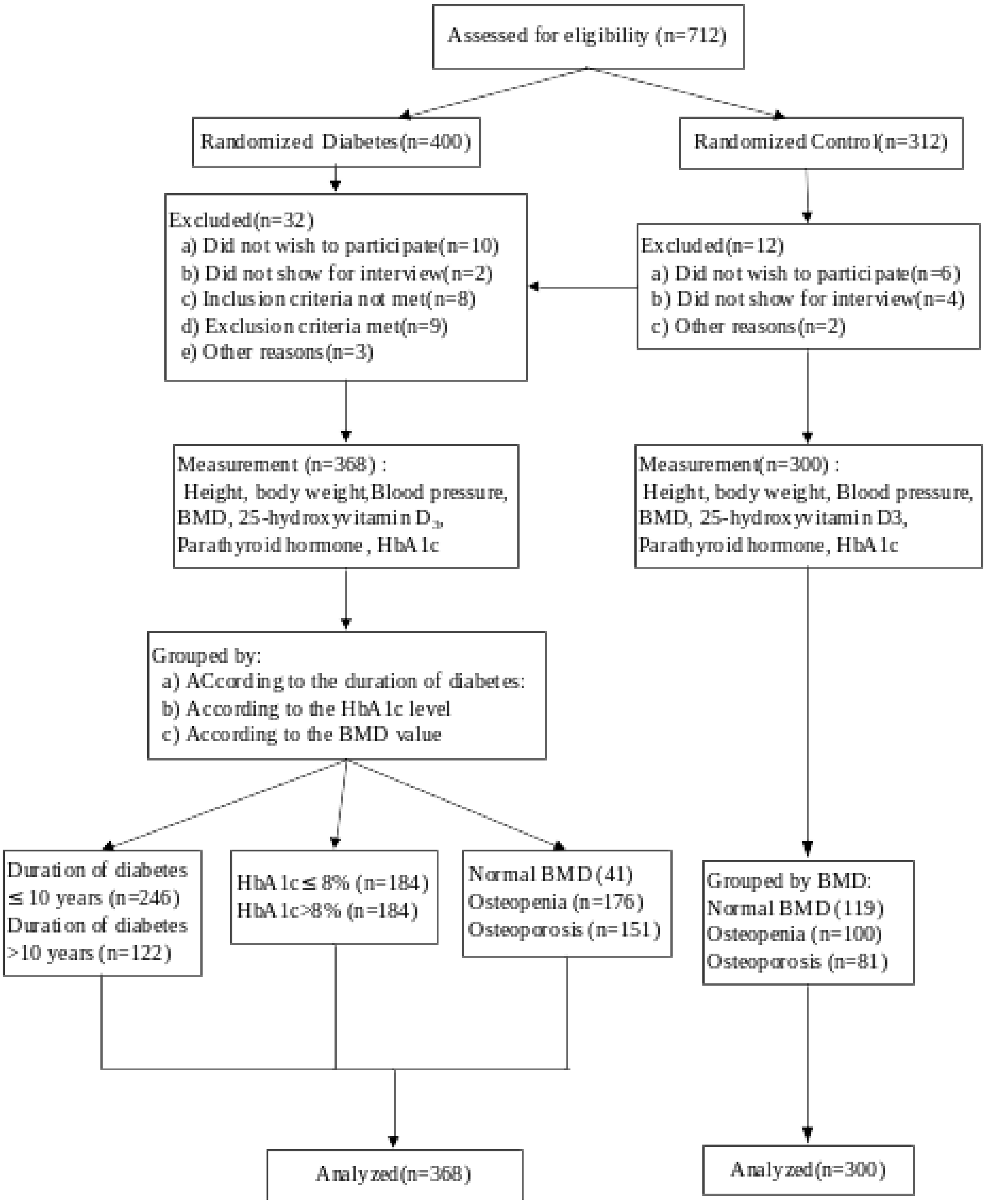

Supplement: Supplementary file 6 [file pone.0188894.s006.docx]
